# Potential targets and molecular mechanisms of D-pinitol against acute kidney injury based on network pharmacology and experimental validation

**DOI:** 10.1080/0886022X.2026.2713919

**Published:** 2026-08-10

**Authors:** Yilin Ren, Yuan Ge, Keyu Zhang, Hui Lin, Dan Niu, Weixia Han, Xiaole Su, Lihua Wang, Xi Qiao

**Affiliations:** a Department of Nephrology, Second Hospital of Shanxi Medical University, Taiyuan, China; b Shanxi Kidney Disease Institute, Taiyuan, China; c Kidney Research Center of Shanxi Medical University, Taiyuan, China; d Department of Nephrology, Xuanwu Hospital Capital Medical University, Beijing, China; e Department of Pathology, Second Hospital of Shanxi Medical University, Taiyuan, China

**Keywords:** D-pinitol, acute kidney injury, apoptosis, network pharmacology, molecular docking, PI3K/AKT signaling pathway

## Abstract

Acute kidney injury (AKI) is a severe clinical syndrome, with ischemia/reperfusion (I/R) being one of its most common causes. Although D‑pinitol (DP), an inositol‑like bioactive molecule, is known to confer renal protection, its efficacy against I/R‑induced AKI remains unknown. A mouse kidney I/R model was employed to evaluate the renoprotective effect of DP. Relevant targets associated with DP and AKI were retrieved from publicly available databases. Subsequently, network pharmacology analysis was conducted to identify the potential targets and signaling pathways. Molecular docking was then performed to predict the binding affinity of DP to core targets identified. Furthermore, *in vivo* and *in vitro* experiments were performed to validate these findings. Systemic toxicity was assessed by serological and histopathological examinations. The results show that DP significantly attenuated I/R**-**induced kidney dysfunction and apoptosis. Network pharmacology analysis identified 108 overlapping targets, with AKT1, HSP90AA1, SRC, CASP3, and MMP9 identified as core targets. Kyoto Encyclopedia of Genes and Genomes (KEGG) enrichment analysis revealed PI3K/AKT signaling pathway as the primary mechanism. Consistent with these predictions, DP enhanced PI3K and AKT phosphorylation in kidney tissue. Molecular docking indicated that DP exhibited the strongest binding affinity to SRC, suggesting it as a potential target. In a hypoxia/reoxygenation (H/R)-induced human renal proximal tubular epithelial (HK-2) cell model, DP significantly increased the phosphorylation of SRC, PI3K, and AKT, and these effects were abrogated by the SRC specific inhibitor PP2. Collectively, DP alleviated I/R‑induced injury and apoptosis, potentially through activation of the SRC/PI3K/AKT signaling pathway.

## LAY SUMMARY

Acute kidney injury (AKI) is a serious and potentially life-threatening condition that currently lacks specific and effective treatments. This study investigated whether D-pinitol (DP)—a natural compound found in soybeans and other legumes—can protect the kidney from damage caused by interrupted blood flow (a condition known as ischemia/reperfusion injury, I/R injury). Using computer-based prediction tools (network pharmacology and molecular docking), we first identified the potential molecular targets and protective pathways of DP. We then confirmed these predictions through laboratory and animal experiments. Our results showed that DP significantly improved renal function and reduced tissue damage after I/R injury. Mechanistically, DP activated the SRC/PI3K/AKT cell survival pathway, which effectively reduced the death of renal tubular cells. As a safe and naturally occurring compound, DP represents a promising new preventive strategy for patients at high risk of developing AKI.

## KEY MESSAGES

1. What is known: Acute kidney injury (AKI) remains a serious clinical syndrome with limited specific therapies, and natural bioactive compounds such as D-pinitol (DP) have shown renoprotective potential in other kidney disease models.
2. This study adds: We demonstrate that DP effectively attenuates ischemia/reperfusion-induced AKI by activating the SRC/PI3K/AKT signaling pathway, thereby reducing renal tubular cell apoptosis and preserving kidney function.
3. Potential impact: These findings provide a mechanistic rationale for DP as a preventive therapeutic strategy for AKI in high-risk clinical settings.

## GRAPHICAL ABSTRACT

**Figure:**
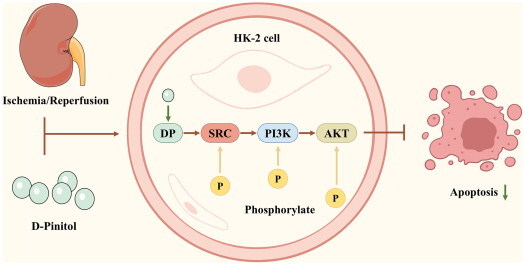

## 1. Introduction

Acute kidney injury (AKI) is a severe clinical syndrome characterized by a rapid decline in renal function. Its global incidence is continuously rising, posing a significant public health challenge [1]. Epidemiological data indicate that the incidence of AKI among hospitalized patients ranges from 10% to 15% [2]. Among critically ill patients requiring renal replacement therapy, the in-hospital mortality rate of AKI is as high as 40%, whereas the 10-year survival rate among survivors is approximately 20% [3]. Clinically, AKI frequently occurs in the context of infections, trauma, invasive procedures, obstructive uropathy, and ischemia/reperfusion (I/R), the latter representing one of the most common causes [4,5]. AKI can progress to chronic kidney disease (CKD) and even end-stage renal disease (ESRD) through multiple pathological mechanisms, including inflammatory cascades, microvascular injury, metabolic reprogramming, and oxidative stress, ultimately leading to irreversible long-term renal function loss [4,6,7]. Histopathological hallmarks include tubular epithelial cell necrosis, brush border loss, luminal dilatation, and interstitial inflammatory infiltration [8]. AKI may also give rise to extra-renal complications, including uremic encephalopathy [9]. Currently, there are no specific effective therapies for AKI beyond supportive care. In patients with severe kidney failure, kidney replacement therapy remains the primary clinical intervention [10]. Given the high mortality and morbidity associated with AKI, the development of novel protective strategies is of paramount clinical significance.

It is noteworthy that natural products have garnered significant attention for their safety, efficacy, and diverse biological activities. They have been used as conventional or complementary agents in healthcare and disease prevention [11]. Traditional Chinese Medicine (TCM), in particular, represents a millennia-old therapeutic system with extensive clinical experience, and is recognized as an essential strategy for the prevention and treatment of various diseases, including kidney disorders [12]. Emerging evidence has demonstrated that TCM-derived phytochemicals exert renoprotective effects against AKI through multi-target and multi-pathway mechanisms, offering unique advantages such as reduced adverse effects and improved clinical outcomes [13]. For instance, rhubarb and its anthraquinone components have been shown to protect against kidney injury by ameliorating gut microbial dysbiosis, restoring aberrant non-coding RNA expression, and correcting metabolite disturbances [12]. Furthermore, natural compounds such as curcumin and sulforaphane have been shown to activate the Keap1/Nrf2 antioxidant pathway and thereby attenuate AKI [14]. Additionally, alginate oligosaccharide, a natural product derived from brown seaweed, has been reported to prevent kidney I/R injury by inhibiting TLR4/MyD88/NF-κB inflammatory signaling cascade and attenuating apoptosis. Collectively, these findings underscore the therapeutic potential of natural products and TCM-derived compounds in AKI management [15].

Among these natural compounds, D-pinitol (DP), derived from soybeans and soy products, has garnered significant attention. DP is a bioactive molecule belonging to the inositol class and its medicinal properties have been extensively investigated. It has demonstrated multiple therapeutic effects, including anti-inflammatory, antioxidant, anticancer, and anti-osteoporosis activities [16]. Additionally, DP has exhibited nephroprotective effects by attenuating cisplatin-induced AKI through the inhibition of oxidative stress and inflammation [17]. Recent studies have also indicated that DP can suppress endoplasmic reticulum stress and apoptosis in I/R-induced organ damage [18]. However, the effects of DP on kidney I/R-induced AKI and the underlying mechanisms remains to be elucidated.

Network pharmacology has emerged as a powerful tool for identifying bioactive compounds and elucidating the potential targets of drugs. Over the past few years, it has been extensively used to evaluate the biological effects and molecular mechanisms of small molecules derived from various natural resources [19]. Molecular docking, on the other hand, is capable of predicting binding affinity and conformation by simulating ligand-target interactions [20]. In the present study, we integrated network pharmacology and molecular docking to screen and analyze key targets and pathways involved in the protective effects of DP against kidney I/R-induced AKI. Our findings were further validated through *in vitro* and *in vivo* experiments. This research aims to elucidate the molecular mechanisms underlying the nephroprotective effects of DP, thereby providing a reliable theoretical and experimental basis for its clinical application and offering a novel approach for the treatment of AKI.

## 2. Materials and methods

### 2.1. Animals

The animal experiments were approved by the Animal Ethics Committee of the Second Hospital of Shanxi Medical University (ethics number: DW2024060). *In vivo* operational procedures, including prophylactic drug administration, AKI modeling, and sample collection, were conducted between January to March 2025. Male C57BL/6J mice (6–8 weeks old, weighing approximately 20–25 g) were provided by Jimei Pharmaceutical Co., Ltd. and housed in a sterile environment at Shanxi Medical University.

### 2.2. Surgeries and groups

Intervention with DP (MCE, HY-N0655, Monmouth Junction, NJ, USA) or distilled water was administered *via* gavage 1 week prior to the induction of I/R injury. Model establishment of kidney I/R: After fasting for 8 h, mice were anesthetized with sodium pentobarbital (50 mg/kg, i.p.). Model establishment of kidney I/R: After fasting for 8 h, mice were anesthetized with sodium pentobarbital (50 mg/kg, i.p.). The bilateral kidney pedicles were clamped for 30 min, followed by a 24h reperfusion period. The sham group underwent the same surgical procedure without clamping the kidney pedicles. Animals were killed through an overdose of pentobarbital sodium following each experiment.

Based on previous literature, mice were randomly assigned to four groups (*n* = 6 per group): sham, I/R, I/R + low-dose DP (I/R + LDP, 50 mg/kg), and I/R + high-dose DP (I/R + HDP, 100 mg/kg) [21]. Blood and kidney samples were collected 24 h after reperfusion for the assessment of kidney function and histopathological analysis.

### 2.3. Histopathological examination

The kidney tissues were fixed in paraformaldehyde, embedded in paraffin, and sectioned at a thickness of 2 µm. The sections were subsequently stained with periodic acid-Schiff (PAS) and hematoxylin and eosin (HE) to evaluate the pathological alterations in the kidney. For tubulointerstitial injury assessment, a scoring system was employed: 0 (no tubulointerstitial damage); 1 (lesion area < 25%); 2 (lesion area 25%-50%); 3 (lesion area > 50%). Ten random fields at 100 × magnification were examined for each specimen, and the mean score was calculated. Staining was analyzed under light microscopy using two independent, blinded observers.

To evaluate the potential off-target effects of DP on other organs, liver tissues were also fixed in 4% paraformaldehyde, embedded in paraffin, sectioned at 4 µm, and stained with HE for histopathological examination under light microscopy. Hepatic morphology was assessed, including hepatocellular integrity, sinusoidal architecture, and the presence of necrosis, steatosis, or inflammatory infiltration.

### 2.4. Immunohistochemistry

Immunohistochemistry for kidney injury molecule-1 (KIM-1) and neutrophil gelatinase-associated lipocalin (NGAL) was performed on the kidney sections using an SP kit, following the manufacturer’s instructions. The sections were stained brown with 3,3-diaminobenzidine and counterstained with hematoxylin. Staining was analyzed under light microscopy using two independent, blinded observers. The collected images were quantitatively assessed using Image J software.

### 2.5. Serum biochemical assays

Kidney function was assessed by measuring serum creatinine (Scr) (Biosino, 100020170, Beijing, China) and Blood Urea Nitrogen (BUN) (Biosino, 100000280, Beijing, China) using commercially available kits, following the manufacturer’s protocols.

To further evaluate the safety profile of DP, serum cardiac troponin T (cTnT) levels were measured using a commercially ELISA kit (Abcam, ab246529, Cambridge, UK) Serum alanine aminotransferase (ALT) and aspartate aminotransferase (AST) activities were determined using commercial assay kits (Biosino, 100020000 and 100020010, Beijing, China). All procedures were performed in accordance with the respective manufacturers’ instructions.

### 2.6. Immunofluorescence staining

Apoptosis was measured using a TUNEL assay kit (Solarbio, T2190, Beijing, China) according to the manufacturer’s instructions. TUNEL-positive cells were counted in four randomly selected fields of view under a fluorescence microscope at 400× magnification. The rate of apoptosis was analyzed using ImageJ software.

### 2.7. Obtaining targets associated with DP and AKI

The standardized SMILES notation for DP was obtained from the PubChem database (https://pubchem.ncbi.nlm.nih.gov/) [22]. This notation was subsequently submitted to the SwissTargetPrediction (http://www.swisstargetprediction.ch/, Probability > 0) [23] and the PharmMapper database (https://lilab-ecust.cn/pharmmapper/) [24] to identify potential molecular targets, with the species restricted to ‘Homo sapiens’. The identified targets were normalized by the UniProt protein database (https://www.uniprot.org/) [25].

To identify targets associated with AKI, the keyword ‘Acute kidney injury’ was queried in the GeneCards database (https://www.genecards.org/, Relevance score ≥10) [26], OMIM database (https://omim.org/) [27] and DisGeNET database (https://www.disgenet.org/) [28]. The final list of AKI-associated targets was generated by removing duplicate entries from the combined results.

### 2.8. Network analysis

To explore the potential mechanisms underlying the effects of DP in AKI, we identified shared targets for drugs and diseases. These shared targets were then visualized using a Venn diagram. The intersecting genes were subsequently uploaded to the STRING database (https://string-db.org/) [29], with the species set to ‘Homo sapiens’ and the ‘minimum required interaction score’ set to 0.400, to construct a protein-protein interaction (PPI) network. The resulting PPI network was visualized using Cytoscape software (version 3.10.0) [30]. The topological properties of the nodes within the network were analyzed using the CytoNCA plugin [31]. In accordance with the methodology established in the previous study, the top 5 genes with the highest degree centrality were identified as crucial hub genes [32].

### 2.9. GO and KEGG pathway enrichment analysis

We performed Gene Ontology (GO) analysis, which was categorized into biological process (BP), cell component (CC), and molecular function (MF), as well as Kyoto Encyclopedia of Genes and Genomes (KEGG) enrichment analyses on common targets for DP and AKI using the DAVID database (https://davidbioinformatics.nih.gov/) [33]. The results were prioritized by adjusted P-value (*p* < 0.05), with the top 30 KEGG pathways and top 10 GO terms selected. GO and KEGG pathway enrichment analysis bubble plots were generated using the Microbiotics Web site (http://www.bioinformatics. com.cn/).

### 2.10. Molecular docking

The 3D structure of DP was retrieved from the PubChem database (https://pubchem.ncbi.nlm.nih.gov/) [22] and utilized as a ligand file for subsequent molecular docking studies. The 3D structures of the core genes were obtained from the UniProt (https://www.uniprot.org/) [25] and PDB (http://www.rcsb.org/) [34] databases and served as protein receptors. Molecular docking was performed using AutoDock Vina. A binding affinity of ≤ −4.0 kcal/mol was considered indicative of effective receptor-ligand interactions, with lower values representing more stable binding [35]. The optimal conformations of the compounds and their interactions with the proteins were analyzed and visualized using PyMOL software.

### 2.11. Real-time qPCR

Total RNA was extracted from kidney tissue samples using TRIzol reagent (Invitrogen) Subsequently, reverse transcription to cDNA was performed using a HighCapacity cDNA Reverse Transcription Kit (Takara Bio Inc., Dalian, China), following the manufacturer’s instructions. The relative mRNA levels were quantified using the 2^−ΔΔCt^ method and normalized to the expression of 18S rRNA. The sequences of the gene-specific primers are listed in Table 1.

**Table 1.** The primer sequence.

Gene	Forward	Reverse
KIM-1	CAGGGAAGCCGCAGAAAA	GAGACACGGAAGGCAACCAC
NGAL	ACAACCAGTTCGCCATGGTA	AGCTCCTTGGTTCTTCCATACAG
AKT1	AGAAGCAGGAGGAGGAGGAG	CCCAGCAGCTTCAGGTACTC
HSP90AA1	AGGAGGTTGAGACGTTCGC	AGAGTTCGATCTTGTTTGTTCGG
SRC	GAACCCGAGAGGGACCTTC	GAGGCAGTAGGCACCTTTTGT
CASP3	TGGGACTGATGAGGAGA	ACTGGATGAACCACGAC
MMP9	GGGACGCAGACATCGTCATC	CGTCATCGTCGAAATGGGC

### 2.12. Cell culture and treatment

Human renal proximal tubular epithelial cells (HK-2, Cell Bank of the Chinese Academy of Sciences, Shanghai, China) were cultured in DMEM (SH30022.01, HyClone, Logan, UT, USA) supplemented with 10% fetal bovine serum (FBS, Solarbio, F8245, Beijing, China) at 37 °C in a humidified atmosphere containing 5% CO_2_. The hypoxia/reoxygenation (H/R) model was established by exposing cells to hypoxic conditions (1% O_2_, 5% CO_2_, 94% N_2_) for 24h, followed by reoxygenation for 6h [36]. DP was first dissolved in distilled water and then diluted in DMEM to achieve the final working concentration. Cells were pretreated with low-dose DP (LDP, 5 μM), high-dose DP (HDP, 10 μM), or high-dose DP combined with the SRC inhibitor PP2 (10 μM, MCE, HY-13805, Monmouth Junction, NJ, USA) for 1h prior to H/R exposure [37,38]. PP2 was first dissolved in DMSO and then diluted in DMEM to achieve the final working concentration. The final DMSO concentration in the culture medium did not exceed 0.1%. Cells in the vehicle control group were treated with an equivalent volume of DMSO diluted in DMEM at the same concentration. The cells were then assigned to the following groups: control, H/R, H/R + LDP, H/R + HDP and H/R + HDP+PP2.

### 2.13. Western blot analysis

Kidney tissues and cultured HK-2 cells were lysed using radio-immuno-precipitation-assay (RIPA) buffer enriched with phosphatase inhibitors and protease inhibitors. Protein concentrations were quantified using a bicinchoninic acid (BCA) assay. Equal amounts of protein were separated by SDS-PAGE and transferred to polyvinylidene fluoride (PVDF) membranes. The membranes were incubated overnight at 4 °C with the following primary antibodies: KIM-1 (1:1000; Abcam, ab314015, Cambridge, UK), NGAL (1:1000; Abcam, ab125075, Cambridge, UK), cleaved caspase-3 (1:500; affbiotech, AF7022, OH, USA), SRC (1: 1000, CST, #2109, Danvers, MA, USA), P-SRC (1:1000, CST, #6943, Danvers, MA, USA), PI3K (1:1000; Abcam, ab191606, Cambridge, UK), P-PI3K (1:1000; Abcam, ab182651, Cambridge, UK), AKT (1:2000; CST, #9272, Danvers, MA, USA), P-AKT (1:2000; CST, #4060, Danvers, MA, USA) and β-Actin (1:10,000; Abclonal, AC026, Wuhan, China). After washing, the membranes were incubated with appropriate secondary antibodies. The protein bands were visualized using the Bio-Rad ChemiDoc MP Imaging System.

### 2.14. Statistical analysis

Statistical analyses were performed using SPSS software (version 22.0). Data are presented as means ± standard error of the mean (SEM). GraphPad Prism was employed for data visualization and plotting. Sample sizes for vivo experiments (*n* = 6 per group) and vitro experiments (*n* = 4 biological replicates) were predetermined based on previous publications with similar experimental designs [21,37], which demonstrated sufficient statistical power to detect significant differences. Animals were randomly assigned to experimental groups. All histopathological assessments, immunohistochemical quantifications, and Western blot densitometric analyses were performed by two independent observers who were blinded to group allocation. Each experiment was repeated at least three times to ensure reproducibility. Comparisons between two groups were analyzed using Student’s t-test. For multiple groups comparisons, one-way analysis of variance (ANOVA) was performed, followed by Tukey’s honestly significant difference (HSD) post-hoc test for pairwise comparisons. Exact P values are provided where applicable, and a *p* < 0.05 was considered statistically significant.

## 3. Results

### 3.1. DP attenuates pathologic injury and protects kidney function in I/R mice

**Figure 1.**
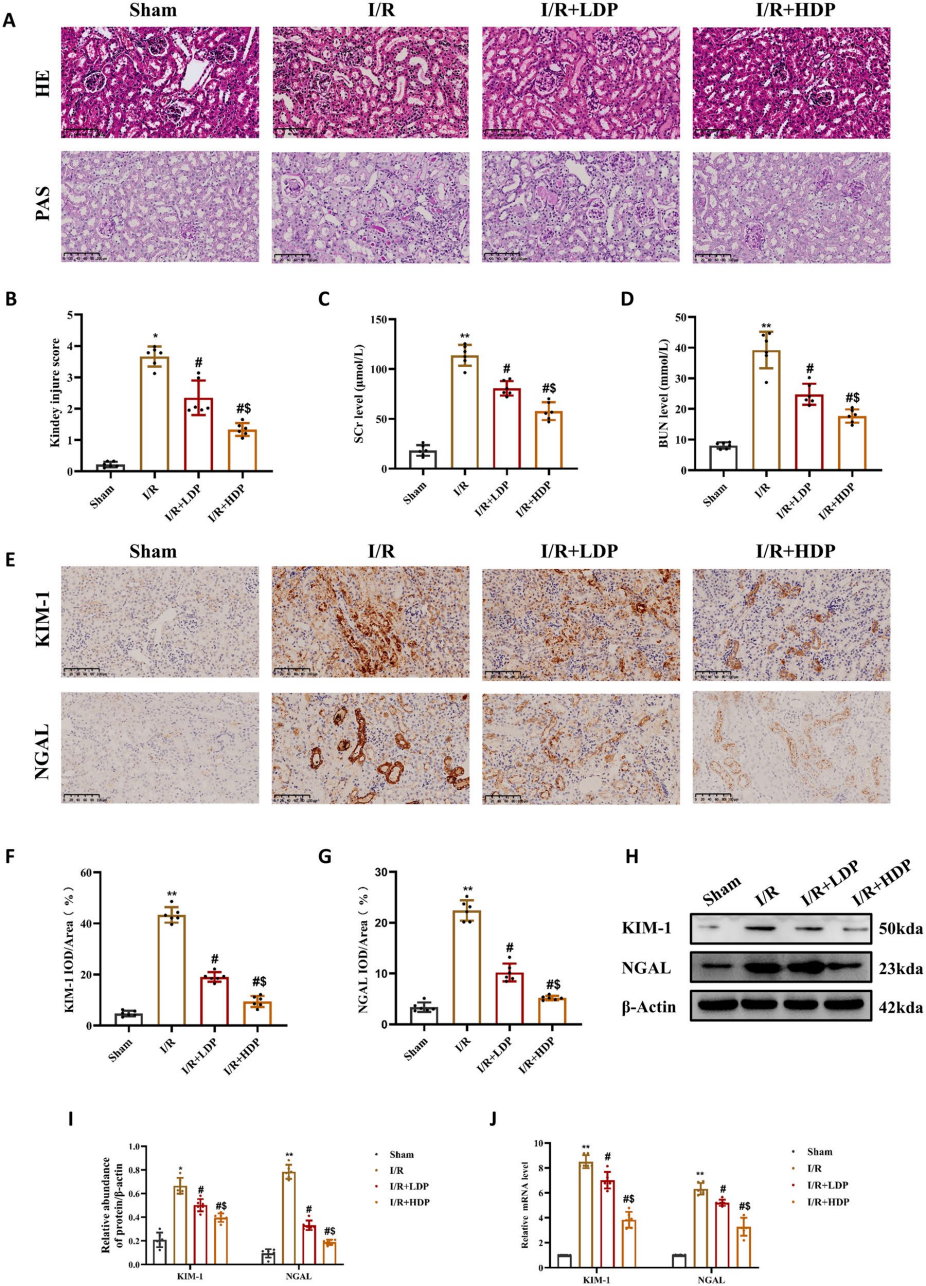
DP protects kidney function and reduces kidney pathology injury in AKI mice. (A) Representative images of kidneys from mice stained with PAS or HE. Scale bar = 100 μm. (B) Tubular injury score assessed by HE staining. (C, D) Quantitative analysis of serum creatinine (SCr) and blood urea nitrogen (BUN). (E) Immunohistochemical images of KIM-1 and NGAL. Scale bar = 100 μm. (F, G) Semi-quantitative analysis of immunohistochemical staining for KIM-1 and NGAL. (H) Western blot (WB) analysis of protein expression levels of KIM-1 and NGAL in kidney tissue. (I) Quantitative analysis of WB results for KIM-1 and NGAL. (J) Quantitative analysis of PCR results for KIM-1 and NGAL. For *in vivo* experiments, *n* = 6 mice per group; for Western blot and qPCR, *n* = 3 independent experiments. All data are shown as mean ± SEM. *p < 0.05 and **p < 0.01 for I/R *vs.* Sham; #*p* < 0.05 for I/R + LDP or I/R + HDP *vs.* I/R; $*p* < 0.05 for I/R + HDP *vs.* I/R + LDP.

To verify the protective effect of DP against kidney I/R injury, an *in vivo* kidney I/R model was established to evaluate kidney function and conduct histopathological examination. HE staining showed increased brush border loss and interstitial congestion in the I/R group compared to the sham group. PAS staining presented similar results. These histopathologic abnormalities were significantly alleviated following treatment with LDP and HDP. Moreover, treatment with HDP resulted in a more pronounced attenuation of these histopathological changes, indicating a superior preventive effect compared to that of LDP (Figure 1A). And kidney injury score was showed in Figure 1B. To assess kidney function, Scr and BUN levels were measured. In the I/R group, both Scr and BUN levels were significantly elevated compared to the control group, indicating kidney dysfunction. However, treatment with LDP and HDP effectively mitigated this increase, with HDP treatment exerting a more pronounced effect (Figure 1C, D).

KIM-1 and NGAL are well-established biomarkers for the assessment of kidney injury [39]. Immunohistochemical analysis showed that KIM-1 and NGAL expression were significantly increased in the I/R group. Treatment with both LDP and HDP treatment ameliorated this alteration, with HDP treatment exerting a more pronounced effect (Figure 1E–G). Similarly, western blotting and real-time qPCR analysis showed that LDP and HDP treatment inhibited the protein and mRNA expression of KIM-1 and NGAL in I/R-induced kidneys, with HDP treatment yielding a more significant effect (Figure 1H–J).

Collectively, these findings indicate that both LDP and HDP effectively attenuate I/R-induced AKI, with HDP exhibiting a superior protective effect compared to LDP.

### 3.2. DP attenuates kidney cell apoptosis in I/R mice

**Figure 2.**
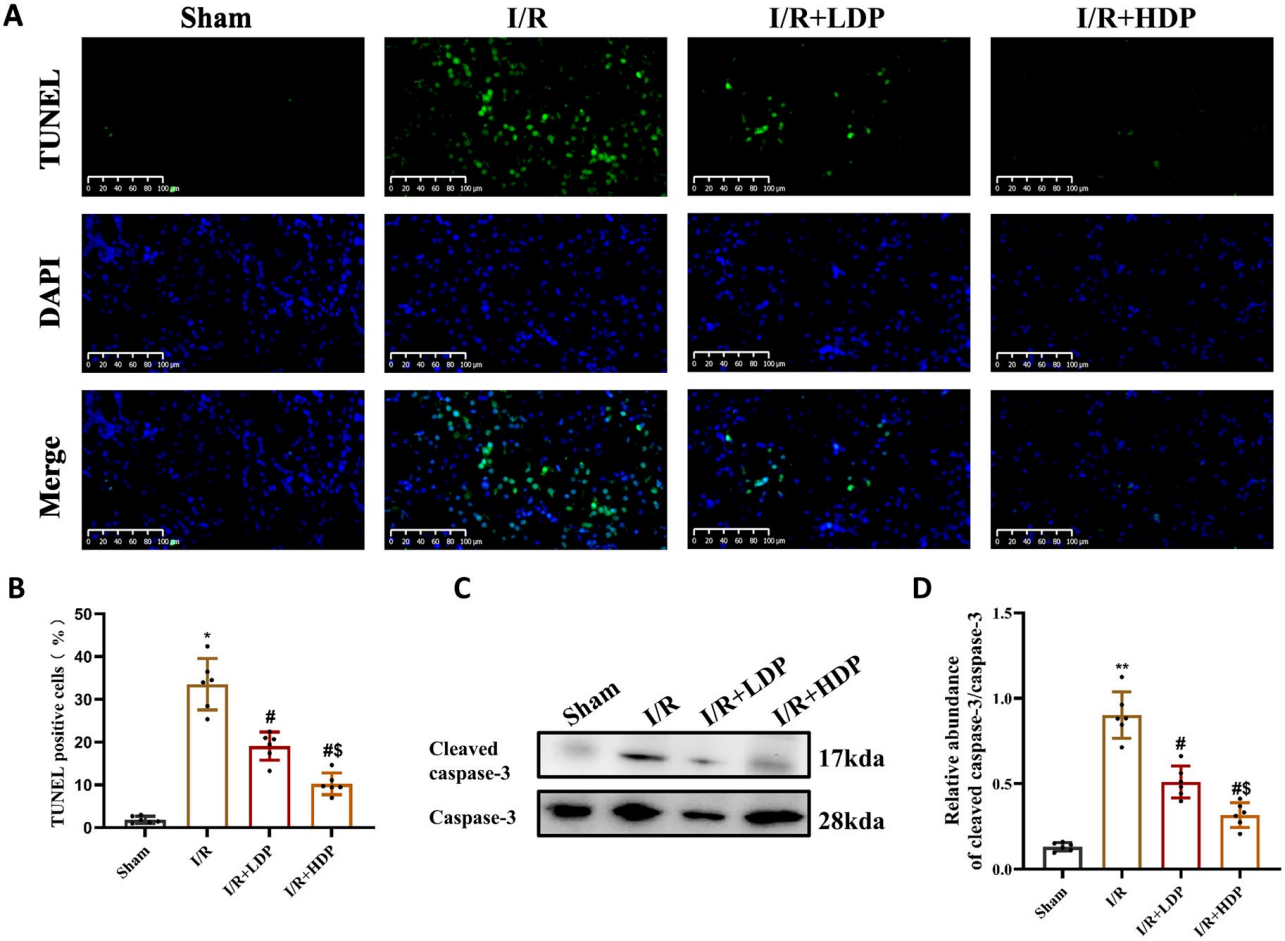
DP attenuates kidney cell apoptosis in I/R mice. (A) TUNEL analysis of kidneys from mice. Scale bar = 100 μm. (B) Quantification of TUNEL-positive cells (green) using ImageJ. Data are presented as the number of TUNEL-positive cells per high-power field (HPF). (C) Western blot (WB) analysis of protein expression levels of cleaved caspase-3 and caspase-3 in kidney tissue. (D) Quantitative analysis of WB results for cleaved caspase-3/caspase-3. For *in vivo* experiments, *n* = 6 mice per group; for Western blot, *n* = 3 independent experiments. All data are shown as mean ± SEM. *p < 0.05, **p < 0.01 for I/R *vs.* Sham; #*p* < 0.05 for I/R + LDP or I/R + HDP *vs.* I/R; $*p* < 0.05 for I/R + HDP *vs.* I/R + LDP.

In comparison with the sham group, the I/R group exhibited a significantly higher incidence of severe kidney cell apoptosis, as evidenced by a higher proportion of TUNEL-positive cells (Figure 2A, B). Following treatment with LDP or HDP, a notable reduction in the TUNEL positive rate was observed, and the HDP treatment further enhancing this effect. Similarly, the I/R group demonstrated an elevated level of cleaved caspase-3 (c-caspase-3). In contrast treatment with LDP or HDP significantly reduced the protein level of c-caspase-3 compared with the I/R group, with a more pronounced reduction observed in the HDP-treated group (Figure 2C, D). The results indicate that DP effectively mitigates kidney cell apoptosis.

### 3.3. Network pharmacology analysis of DP against AKI

**Figure 3.**
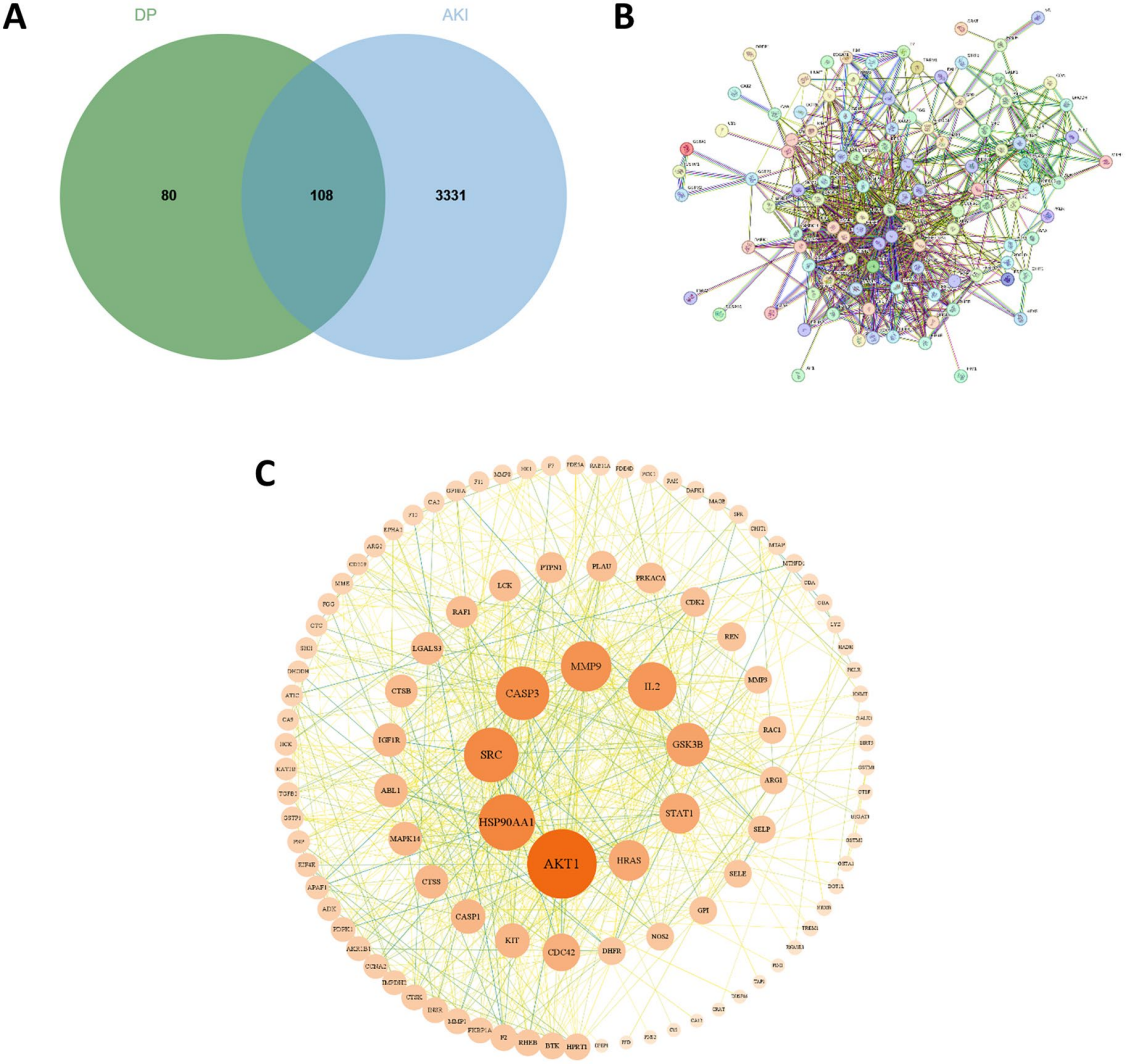
Network Pharmacology Analysis of DP against AKI. (A) Venn diagram of potential targets for DP against AKI. (B) PPI network of shared targets. Each bubble represents a gene protein, and the node’s 3D structure displays the protein’s spatial organization structure. The strength of data support is displayed by the breadth and strength of the lines connecting nodes, reflecting the correlation between various targets. (C) Visualization of the PPI network of the intersecting targets of DP and AKI.

To investigate potential molecular targets of DP for the treatment of AKI, a network pharmacology analysis was performed (Figure 3). The standard SMILES for DP was obtained in the Pubchem database as COC1[C@@H]([C@H](C([C@@H]([C@@H]1O)O)O)O)O. Upon importing the SMILES into the SwissTargetPrediction and PharmMapper databases, a total of 188 corresponding targets for DP were identified (Table S1). Meanwhile, the GeneCards, OMIM and DisGeNET databases yielded a total of 3439 AKI-related target genes (Table S2). By intersecting the targets of DP and AKI, 108 possible targets of DP against AKI were pinpointed (Figure 3A and Table S3). These targets were subsequently uploaded to the STRING platform to construct PPI networks (Figure 3B), which were then visualized using Cytoscape software. Based on the degree values, AKT1, HSP90AA1, SRC, CASP3 and MMP9 emerged as the core targets (Figure 3C).

### 3.4. GO and KEGG analysis

**Figure 4.**
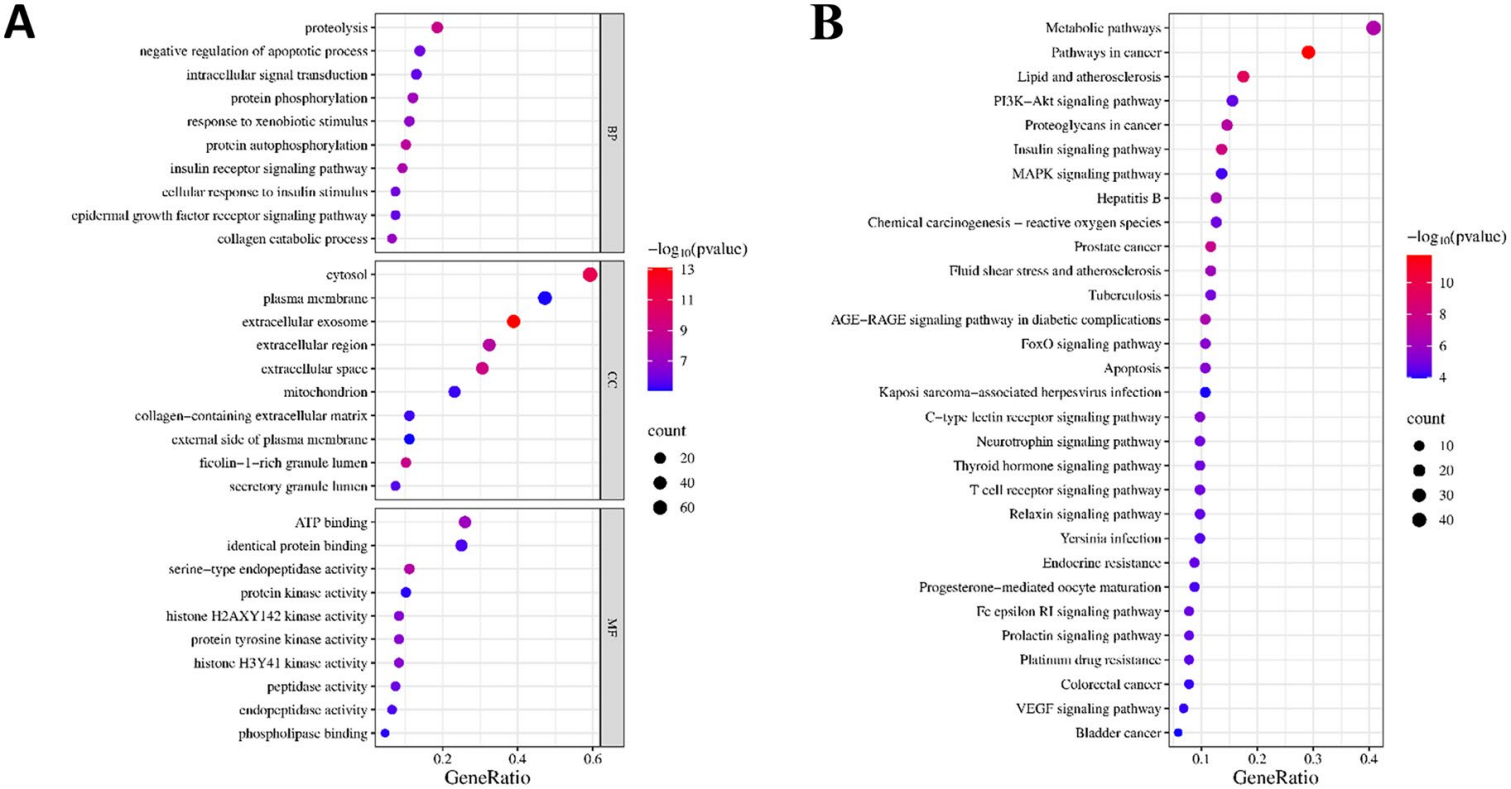
GO and KEGG analysis. (A) The bubble diagram of GO enrichment analysis of DP-AKI genes, including the top 10 significant enrichment terms of BP, CC, and MF. The larger size of a dot indicates the larger number of genes annotated in the entry, and the redder color of a dot stands for the lower the p value. (B) Visualization network of KEGG enrichment pathways. The magnitude and chromaticity of each node symbolize the extent of enrichment of the corresponding KEGG pathway.

The results of GO enrichment analysis unveiled several key BPs, including protein hydrolysis, cell proliferation responses, and proteolysis. CCs were significantly enriched in extracellular matrix, extracellular exosomes, and plasma membrane. As for MFs, protein tyrosine kinase activity, protein binding, and ATP binding were highlighted as important ones (Figure 4A and Table S4). The KEGG pathway analysis, ranked by P-value, showed the top 30 signaling pathways in Figure 4B and Table S5. Notably, the PI3K/AKT signaling pathway, apoptosis pathway and MAPK signaling pathway identified as being associated with the mechanism.

### 3.5. Experimental assessment of the effect of DP on core genes and signaling pathways predicted by network pharmacology

**Figure 5.**
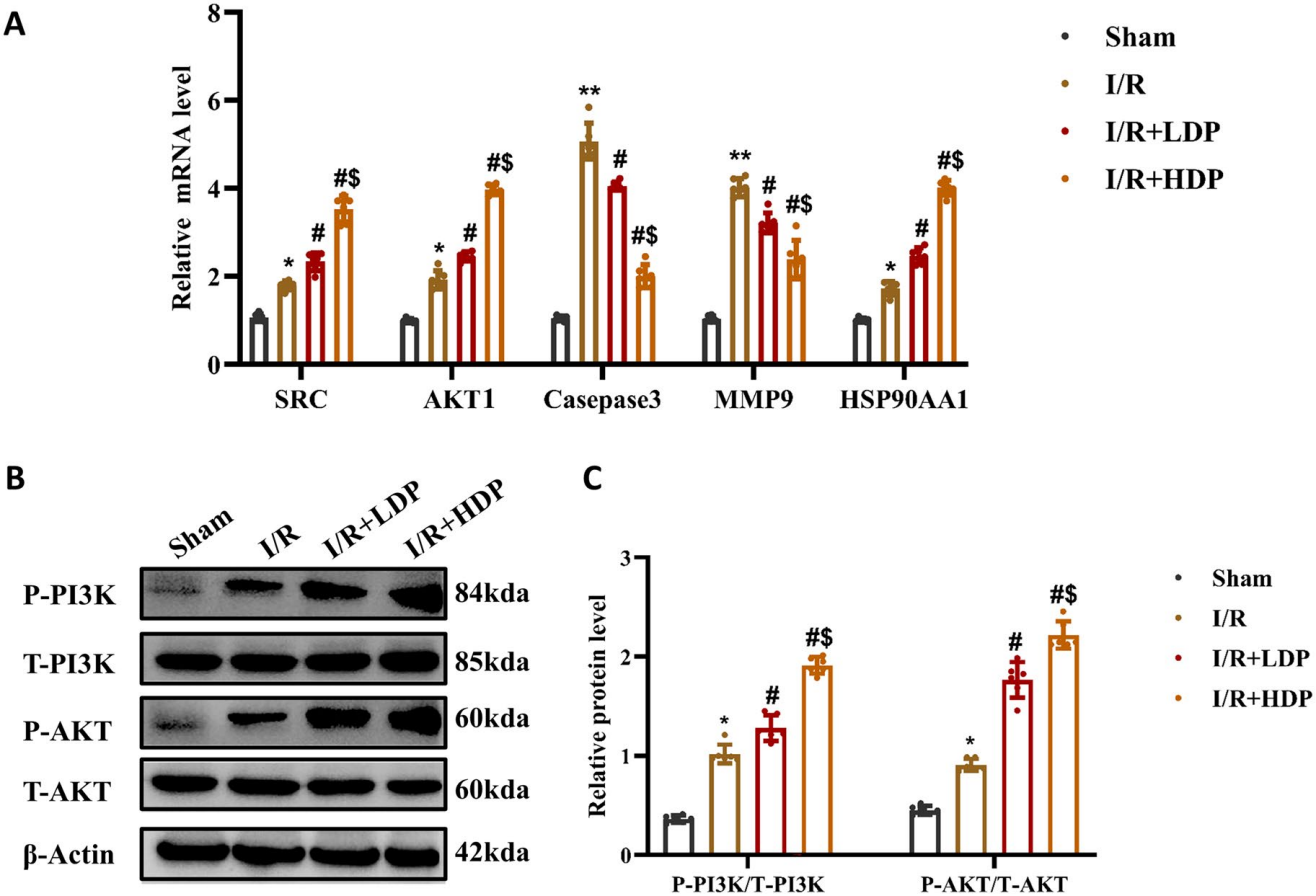
Experimental assessment of the effects of DP on core genes and signaling pathways. (A) Quantitative analysis of PCR results for AKT1, HSP90AA1, SRC, CASP3 and MMP9. (B) Western blot (WB) analysis of protein expression levels of p-PI3K, PI3K, p-AKT, and AKT in kidney tissue. (C) Quantitative analysis of WB results for p-PI3K/PI3K and p-AKT/AKT. For *in vivo* experiments, *n* = 6 mice per group; for qPCR and Western blot, *n* = 3 independent experiments. All data are shown as mean ± SEM. *p < 0.05, **p < 0.01 for I/R *vs.* Sham; #*p* < 0.05 for I/R + LDP or I/R + HDP *vs.* I/R; $*p* < 0.05 for I/R + HDP *vs.* I/R + LDP.

Through meticulous PPI analysis, we scrutinized the mRNA expression profiles of 5 core genes by RT-qPCR. All 5 core genes were significantly upregulated in the I/R group compared with the Sham group (Figure 5A). Treatment with LDP or HDP significantly attenuated the I/R-induced upregulation of CASP3 and MMP9, with the HDP exhibiting a more pronounced inhibitory effect. Conversely, DP pretreatment further elevated the mRNA levels of AKT1, HSP90AA1, and SRC, and this increase was more marked in the HDP-treated group (Figure 5A).

These alterations were effectively reversed by pretreatment with either LDP or HDP, with the HDP-treated group demonstrating more pronounced changes. The results suggest that these pivotal targets may play biological roles in AKI and that DP has a regulatory effect on them.

Furthermore, integrating KEGG analyses into our investigation, we hypothesized that the activation of the PI3K/AKT pathway by DP might serve as a primary protective mechanism against AKI. To validate this hypothesis, we meticulously examined the expression and phosphorylation levels of key proteins within the PI3K/AKT signaling pathway. As depicted Figure 5B, C, the total expression levels of PI3K and AKT remained relatively stable compared to sham group. Yet, a marked increase in their phosphorylation levels was observed after 24h of reperfusion. Notably, treatment with LDP or HDP further elevated the levels of P-PI3K and P-AKT, with the HDP-treated group demonstrating more pronounced changes. Collectively, these results suggest that DP’s ameliorative effects on kidney injury are intricately linked to the activation of the PI3K/AKT pathways, shedding new light on its therapeutic potential in AKI management.

### 3.6. Molecular docking visualization

**Figure 6.**
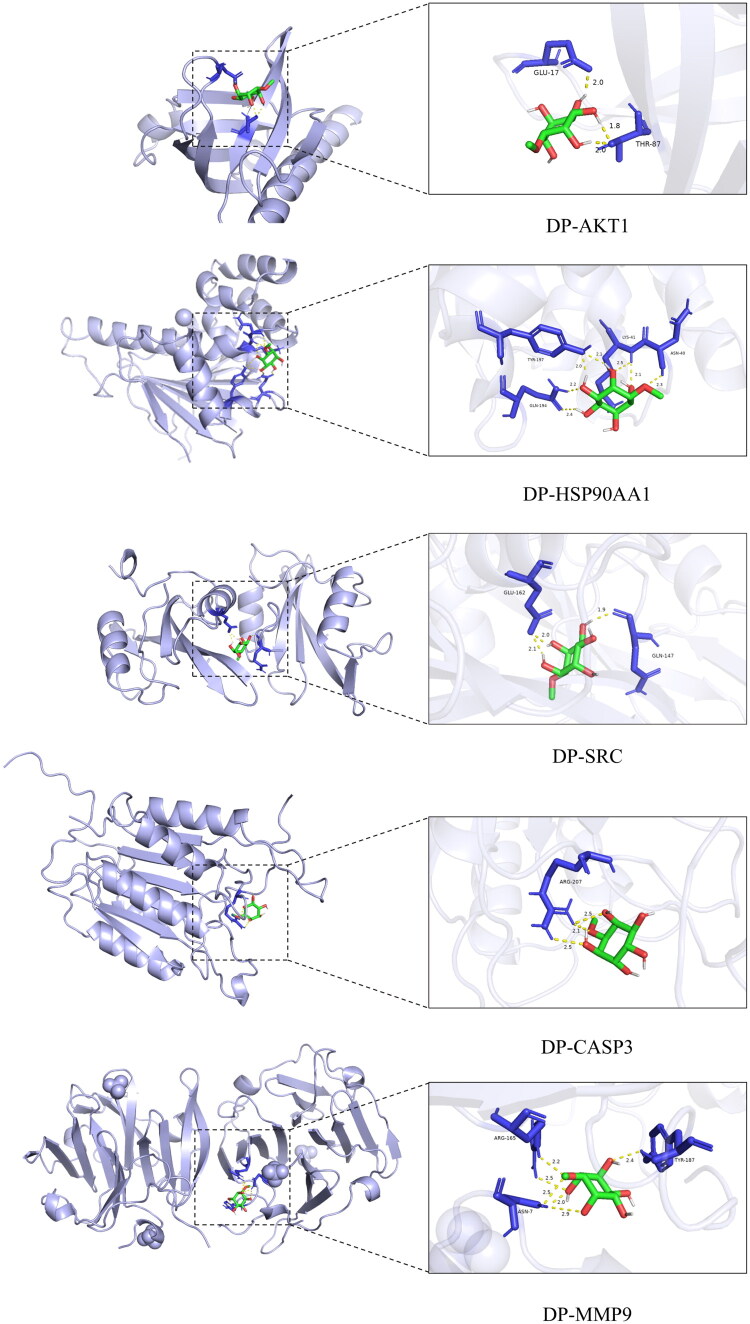
Docking patterns of DP and core genes. Visualization of the docking results between DP and AKT1, HSP90AA1, SRC, CASP3 and MMP9. The binding sites are connected by yellow hydrogen bonds, and the length of the hydrogen bonds is indicated next to the bond.

To determine the binding capacity of DP to core proteins, molecular docking was employed to forecast the binding energy of receptor-ligand complexes. DP was capable of forming stable complexes with all 5 protein receptors. Specifically, DP could form three hydrogen bonds with amino acid residues of AKT1, SRC and CASP3, six hydrogen bonds with MMP9 and seven hydrogen bonds with HSP90AA1 (Figure 6). Molecular docking analysis revealed that DP exhibited the strongest binding affinity for SRC, with the lowest binding energy among all core targets (Table 2 and Table S6). This in silico prediction suggests that SRC is a putative molecular target of DP, however, further experimental validation is warranted to confirm direct physical binding.

**Table 2.** Binding affinities of DP to core targets.

Drug	Target	Minimum binding energy (kcal/mol)
DP	AKT1	−5.4
	HSP90AA1	−5.1
	SRC	−6.0
	CASP3	−4.8
	MMP9	−5.5

### 3.7. DP attenuates H/R-stimulated HK-2 cells apoptosis by activating the SRC/PI3K/AKT pathway

Based on the molecular docking and KEGG enrichment analysis results, we hypothesized that SRC mediates DP-mediated activation of the PI3K/AKT pathway, representing a primary mechanism underlying the renoprotective effects of DP against AKI. To determine whether the protective effect of DP is indeed mediated by SRC, HK‑2 cells were pretreated with PP2, a specific SRC inhibitor, and subsequent changes in downstream signaling and apoptosis were assessed.

**Figure 7.**
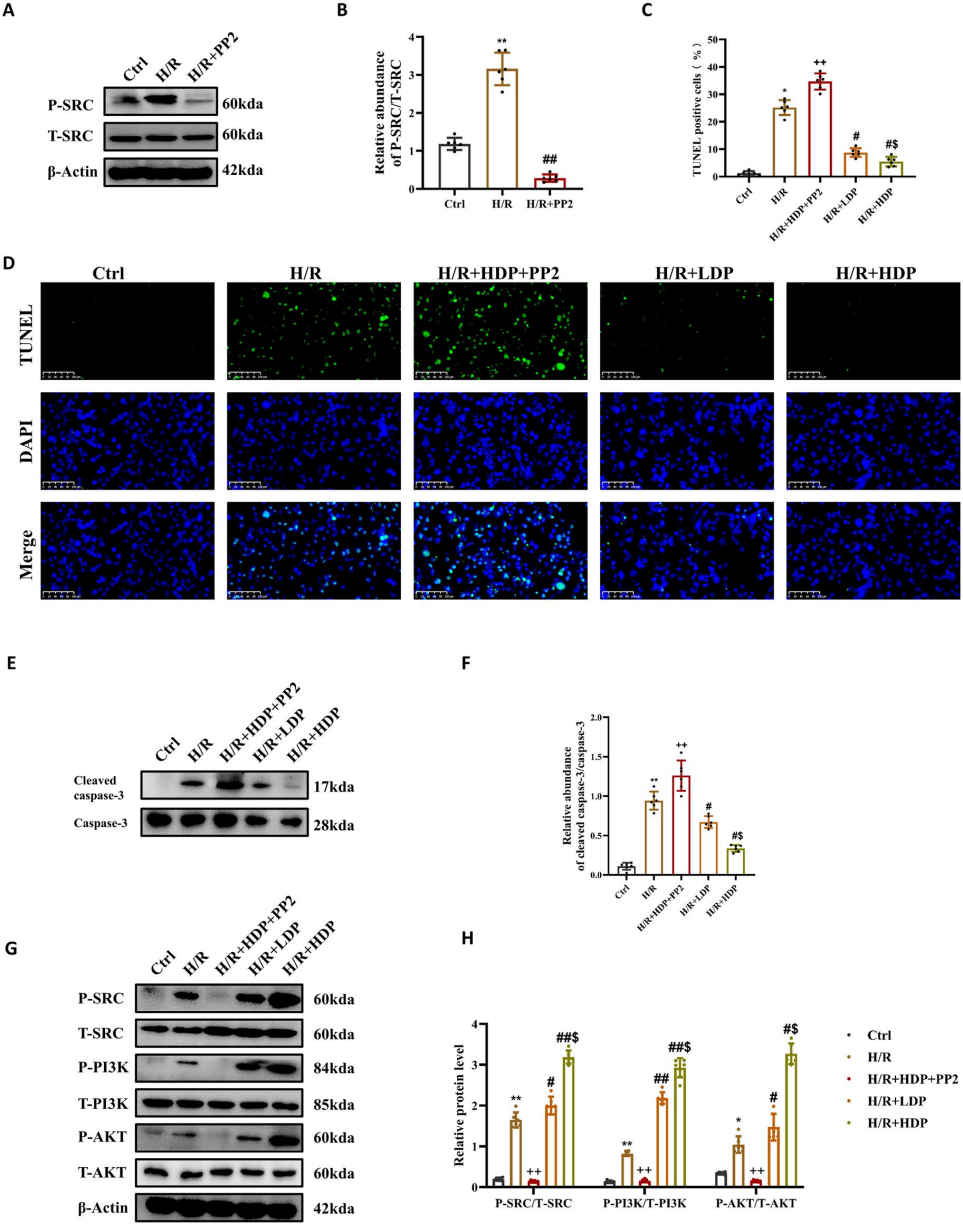
DP attenuates H/R-stimulated HK-2 cells apoptosis by activating the SRC/PI3K/AKT pathway. (A) Western blot (WB) analysis of protein expression levels of p-SRC and SRC in HK-2 cells. (B) Quantitative analysis of WB results for p-SRC/SRC in HK-2 cells. (C) Quantification of TUNEL-positive cells (green) using ImageJ. Data are presented as the number of TUNEL-positive cells per high-power field (HPF). (D) TUNEL analysis of HK-2 cells. Scale bar = 100 μm. (E) WB analysis of protein expression levels of cleaved caspase-3 and caspase-3. (F) Quantitative analysis of WB results for cleaved caspase-3/caspase-3. (G) WB analysis of protein expression levels of p-SRC, SRC, p-PI3K, PI3K, p-AKT, and AKT. (H) Quantitative analysis of WB results for p-SRC/SRC, p-PI3K/PI3K, and p-AKT/AKT. For *in vitro* experiments, *n* = 4 biological replicates per group; for Western blot, *n* = 3 independent experiments. All data are shown as mean ± SEM. *p < 0.05, **p < 0.01 for H/R *vs.* Ctrl; #*p* < 0.05 for H/R + LDP or H/R + HDP *vs.* H/R; $*p* < 0.05 for H/R + HDP *vs.* H/R + LDP; ++*p* < 0.01 for H/R + HDP + PP2 *vs.* H/R + HDP; ##*p* < 0.01 for H/R + PP2 *vs.* H/R.

Western blot experiments were conducted to confirm its inhibitory effect on SRC. As shown in Figure 7A, B, PP2 significantly attenuated the upregulation of P-SRC in the H/R group, while T-SRC levels remained largely unaffected.

The TUNEL assay results were consistent with those observed in kidney tissues, and LDP or HDP effectively ameliorated H/R-stimulated apoptosis in TECs, with a more pronounced effect observed for HDP. Moreover, the protective effect of HDP was significantly diminished when SRC was inhibited (Figure 7C, D). Similarly, the expression levels of c-caspase-3 were inhibited by LDP or HDP and further suppressed by HDP. However, this trend was reversed upon the addition of PP2 (Figure 7E, F). We examined the expression levels and phosphorylation status of SRC, PI3K and AKT in 5 groups (Figure 7G, H). Following H/R injury, the phosphorylation levels of SRC, PI3K, and AKT were significantly elevated in HK-2 cells. Treatment with LDP or HDP further enhanced this effect. Conversely, the inhibition of SRC by PP2 reduced the expression levels of P-SRC/T-SRC, P-PI3K/T-PI3K and P-AKT/T-AKT compared with HDP treatment alone. These findings suggest that DP may exert its anti-apoptotic effects in HK-2 cells by modulating the SRC-mediated PI3K/AKT signaling pathway.

### 3.8. DP does not induce detectable cardiotoxicity or hepatotoxicity

To evaluate potential off-target toxicity of DP at the administered doses, serum biomarkers of cardiac and hepatic injury were assessed. As shown in Figure S1, serum cTnT, ALT, and AST levels did not differ significantly between the I/R and Sham group (*p* > 0.05 for all parameters). Similarly, these markers remained unchanged in the I/R + LDP and I/R + HDP groups compared with the I/R group. Histopathological examination of liver sections (Figure S1) revealed preserved hepatic architecture with intact hepatocytes, clear sinusoidal spaces, and no evidence of necrosis, steatosis, or inflammatory infiltration across all treatment groups, findings comparable to those in the Sham group. These results suggest that, at the doses and treatment duration used in this study, DP does not induce detectable cardiotoxicity or hepatotoxicity.

## 4. Discussion

This study indicate that DP markedly attenuates kidney I/R-induced AKI and curtails kidney cells apoptosis. Subsequent network pharmacology and gene enrichment analyses identified the PI3K/AKT cascade as the principal pathway through which DP confers renoprotection. Molecular docking studies further suggested SRC as the direct molecular target of DP. Corroborated by complementary in-vitro and in-vivo data, these results establish that DP mitigates kidney I/R injury through activation of the SRC/PI3K/AKT signaling pathway.

Kidney I/R injury is the leading cause of AKI in diverse clinical contexts, including major surgery, hemorrhagic shock, and cardiorenal syndrome [40]. Subsequent to I/R, a self-propagating cycle of inflammation and oxidative stress drives kidney damage [41], in severe cases, AKI may progress to chronic kidney disease (CKD), characterized by relentless interstitial fibrosis and irreversible loss of kidney function. Despite decades of research, no targeted therapy has yet been translated into routine clinical practice. DP is a naturally occurring bioactive molecule that has garnered increasing attention for its potent anti-inflammatory and antioxidant activities. Although emerging evidence indicates renoprotective effects of DP confers in models of cisplatin-induced AKI and kidney fibrosis [17,42], its protective efficacy against I/R-induced AKI and the underlying molecular involved remain undefined. In a murine model, we confirmed that DP significantly attenuated I/R-induced histological injury, renal dysfunction, and cell apoptosis. Notably, high-dose DP produced more pronounced improvements in tubular integrity and reduction of apoptotic indices, suggesting higher dose may confer better renoprotective effect. Although the protective effects of low-dose DP were statistically significant, they remained relatively modest, possibly reflecting the rapid and multifaceted pathophysiology of I/R injury that exceeds the capacity of a single pharmacological agent to fully ameliorate.

Systematic mining of multiple public databases identified 108 targets shared between DP and AKI. Network topology and interaction analyses subsequently designated AKT1, HSP90AA1, SRC, CASP3 and MMP9 as the core targets through which DP exerts its renoprotective actions. KEGG pathway enrichment subsequently identified the PI3K/AKT signaling pathway as the principal signaling axis mediating the renoprotective effects of DP. The PI3K/AKT pathway is well established as a central mediator of cell survival that counters apoptosis and mitigates I/R injury [35]. In our *in vivo* AKI model, I/R injury triggered the phosphorylation of PI3K/AKT. However, this response represented a limited and transient endogenous compensatory stress response that was insufficient to effectively counteract I/R-induced apoptosis and oxidative damage. Indeed, despite elevated PI3K/AKT phosphorylation in the I/R group, severe kidney injury and extensive apoptosis persisted. Following DP pretreatment, phosphorylation of PI3K and AKT were further significantly enhanced, concomitant with marked reductions in kidney injury biomarkers and apoptotic markers. These findings suggest that DP transformed inadequate endogenous compensation into robust protective activation capable of attenuating I/R-induced injury. This amplification of protective signaling closely aligns with the classical mechanism of ischemic preconditioning. Importantly, PP2-mediated inhibition of SRC completely abolishes the protective effects of D-Pinitol, demonstrating that the enhanced phosphorylation is mechanistically required for its therapeutic action rather than merely an epiphenomenon of cellular stress. Thus, DP pretreatment may be regarded as a form of pharmacological preconditioning that fortifies this pathway in advance, thereby enabling more effective cytoprotection upon subsequent I/R exposure.

Molecular-docking simulations revealed that DP forms the most stable complex with SRC, displaying the lowest binding energy among all core targets. SRC, a non-receptor tyrosine kinase critical for signal transduction, has been implicated in AKI pathophysiology; its activation attenuates downstream inflammatory and apoptotic cascades in murine AKI [43,44]. These computational and pharmacological data collectively suggest that SRC may mediate the protective effects of DP, although direct physical binding between DP and SRC remains to be experimentally validated. SRC is a well-established upstream activator of the PI3K/AKT signaling pathway, its overexpression potently induces PI3K/AKT pathway and enhances cell survival [45]. To investigate whether the anti-apoptotic effect of DP in AKI is exerted through the SRC/PI3K/AKT signaling pathway, we subjected HK-2 cells to H/R injury. Pretreatment with the SRC kinase-specific inhibitor PP2 markedly suppressed SRC activity and concurrently abrogated DP-induced PI3K/AKT activation and cytoprotection. Collectively, these data demonstrate that DP attenuates I/R-induced AKI, at least in part, by inhibiting TECs apoptosis *via* the SRC/PI3K/AKT signaling pathway. In addition to AKT1 and SRC, network pharmacology identified HSP90AA1, CASP3, and MMP9 as core nodes. Among these, CASP3 serves as a classical executioner of apoptosis, our *in vivo* and *in vitro* data consistently demonstrated that DP significantly reduced cleaved caspase-3 expression, confirming its anti-apoptotic effect. HSP90AA1, a molecular chaperone, has been reported to stabilize AKT and promote its phosphorylation, thereby establishing indirect crosstalk with the PI3K/AKT pathway [35]. MMP9, a matrix metalloproteinase involved in extracellular matrix remodeling and inflammatory cell infiltration, is upregulated in AKI and has been shown to be regulated by PI3K/AKT signaling in certain pathological contexts. Collectively, the combination of network topology and pathway enrichment provides a robust theoretical framework for subsequent experimental validation.

Recent multi-omics Mendelian randomization, machine learning, and network pharmacology studies have systematically interrogated the molecular landscape of AKI, identifying candidate targets [46–48]. Collectively, these findings highlight the value of computational prioritization strategies for uncovering actionable AKI targets. However, we acknowledge the inherent limitations of network pharmacology and database-based target prediction. Although valuable for hypothesis generation, network pharmacology cannot replace functional validation. The AKI-related targets retrieved from GeneCards, OMIM, and DisGeNET encompass a broad spectrum of inflammation-, apoptosis-, and oxidative stress-related genes, many of which are not AKI-specific. Therefore, the core genes selected based on degree centrality represent topologically important nodes rather than functionally validated mediators. To mitigate this limitation, we employed a multi-layered filtering strategy combining PPI topology, KEGG enrichment, and literature-based biological plausibility. Consistent with recent studies that have employed integrated multi-omics and network-based approaches to identify AKI candidate targets followed by experimental validation [46–48], we recognize that computational predictions require orthogonal experimental validation to establish functional relevance. Future investigations employing gene knockdown or overexpression, surface plasmon resonance (SPR), isothermal titration calorimetry (ITC), or cellular thermal shift assay (CETSA) are warranted to definitively confirm the physical binding of DP to targets such as SRC and to elucidate the underlying causal mechanisms.

Notably, various natural products have been reported to exert protective effects against I/R-induced AKI through distinct mechanisms. For instance, rhubarb and its anthraquinone components (e.g., rhein, emodin) ameliorate renal dysfunction and fibrosis by inhibiting the TGF-β/Smad pathway or p53-mediated apoptosis [12]. Curcumin attenuates renal I/R injury through multiple pathways, including modulation of the semaphorin-plexin axis, upregulation of APPL1, and stabilization of mitochondrial function [13,14]. Sulforaphane primarily activates the Nrf2-dependent phase II enzyme system, thereby enhancing antioxidant enzyme expression [14]. Alginate oligosaccharide acts by inhibiting TLR4/MyD88/NF-κB inflammatory signaling and apoptosis [15]. In contrast, DP exhibits a distinctive mechanism *via* activation of the SRC/PI3K/AKT pathway, suggesting that it may serve as a complementary therapeutic option alongside existing natural products for AKI management.

However, several limitations of this study warrant being acknowledged. Firstly, we did not include a DP-only control group, so we could not assess the effects of DP on normal kidney tissue. Although its safety has been demonstrated in preclinical and clinical studies, with no toxicity in mammalian models [16], further cytotoxicity testing would provide more comprehensive safety data. Secondly, our experiments were confined to *in vitro* and animal models, thus, the clinical applicability of our findings remained unverified. Subsequent human trials are essential to validate the efficacy of DP and determine the optimal dosing regimen. Thirdly, our study focused solely on acute injury models. Emerging evidence has shown that DP also exhibits protective efficacy against chronic tubulointerstitial fibrosis progression [42]. Future research could further explore the potential of DP for preventing progression of AKI to chronic kidney disease, thereby contributing to more effective treatment of kidney disease. Fourthly, the prophylactic administration protocol used in this study does not address the clinically relevant scenario of therapeutic intervention after AKI onset. In this study, we adopt a 7-day DP pretreatment regimen, which minimizes interference from post-ischemic cascades and thereby allows clearer elucidation of DP’s direct effects on the PI3K/AKT axis. This design is justified for preliminary mechanistic exploration and mimics prophylactic use in high-risk patients (e.g., patients scheduled for major cardiovascular surgery, transplantation, or contrast-enhanced imaging). Nevertheless, future studies evaluating post-injury administration are warranted to comprehensively assess the therapeutic potential of DP. Additionally, we did not directly measure classical inflammatory cytokines or oxidative stress biomarkers. Although DP is known for its anti-inflammatory and antioxidant activities, and the PI3K/AKT pathway can crosstalk with NF-κB and Nrf2/ARE signaling, our data primarily support its anti-apoptotic effects; the anti-inflammatory and antioxidant contributions remain to be clarified in future studies using targeted inhibitors and corresponding biomarker assessments. Finally, we acknowledge the absence of a positive drug control group in our experimental design. Currently, no FDA-approved pharmacotherapies are specifically indicated for the prevention or treatment of AKI [49,50], and the standard of care remains largely supportive [51]. This is particularly true for I/R-induced AKI, for which no effective pharmacological therapy is currently available [52]. Therefore, we were unable to include positive control in the present study. Instead, we established a dose-response comparison to evaluate the protective efficacy of DP. Future studies may consider incorporating clinically validated positive controls once such agents become available.

## 5. Conclusion

In conclusion, the present study elucidated the potential mechanisms of DP against I/R-induced AKI through an integrated strategy combining network pharmacology, molecular docking, and *in vitro* and *in vivo* experimental validation. Our findings demonstrate that DP attenuates renal I/R injury by suppressing tubular epithelial cell apoptosis *via* activation of the SRC/PI3K/AKT pathway. These results provide novel mechanistic insights into the therapeutic potential of DP for the prophylactic treatment of AKI.

## Supplementary Material

Supplementary tables (1).zip

## Data Availability

The data that support the findings of this study are openly available in Mendeley Data at http://doi.org/10.17632/djkv32m843.1, reference number djkv32m843.1.
